# Notes and News

**Published:** 1891-12-12

**Authors:** 


### NOTES AND NEWS.

COLLECTED AND COMMUNICATED.

THE third general meeting of the Hospitals Association will be held on Wednesday, the 16th inst., in the Court Room at Guy's Hospital, when Mr. G. A. Cross will read a paper on "Should Charities Pay Rates." Mr. G. N. Lushington (Treasurer of Guy's Hospital) will take the chair at eight p.m.

MR. CHARLES BOOTH will read a paper on "Enumeration and Classification of Paupers," and "State Pensions for the Aged," at 28, Jermyn Street, S.W., on the 15th inst., at a quarter to eight p.m.

WE are glad to hear that the Committee of the Poplar Hospital for Accidents have decided to immediately extend their buildings, the need for which is most urgent. Two new wards, and 40 additional beds, will be provided, with a new out-patient department, and more suitable accommodation for the staff. It is greatly to be regretted that the Committee have had to sell out a considerable amount of their funded capital to enable building operations to be commenced. The hospital is in the heart of a district where accidents occur almost daily, and the nearest general hospital is two miles distant. The institution has an excellent record, one of the best in London; many of its patients are homeless sailors. At least ten thousand pounds will be required, and we hope loyal and patriotic hearts will provide all the money within the next few months.

THE foundation stone of the New Home for Incurables, which is being built by the Newcastle-upon-Tyne Corporation, as trustees of the Hospital of St. Mary Magdalene, was laid on the 22nd inst., by Mrs. Thomas Richardson, wife of Alderman Thomas Richardson, J.P., Chairman of the Schools and Charities Committee. In the old Home for Incurables, which is situated in the midst of pleasant grounds, there is only accommodation for thirty patients, and the inadequacy of this is the cause for the new hospital which is being built in the same grounds. In the new building there will be accommodation for sixty-eight patients on the semi-pavilion system with an administrative block for the Matron and servants. Twenty of the patients admitted will be sufferers from cancer for whom a distinct pavilion will be reserved—with a connecting corridor on the ground floor only. The corridor and staircases throughout will be of fire-proof construction. The main corridors spacious, well lighted, and warmed, will serve as ambulatories for the patients in inclement weather. The building will be on a raised terrace commanding a view of the grounds, which are already nicely laid out, and will be of a brick faced with pressed red brick, dark red Normandy quoins to windows, Prudham stone dressings. Messrs. Middlemass Brothers are the contractors, and Mr. Edward Shewbrooks, F.R.I.B.A., the architect.

THE question of sterilising water for the supply of cities by means of electricity has again cropped up in a paper by Mr. R. Meade Bache, recently read before the American Philosophical Society. Mr. Bache has made a number of experiments, which go to prove that a current of electricity sent through water destroys bacteria; but, as in prior experiments by others, it is still doubtful whether the liberated oxygen or the electricity itself kills the germs. In any case the water is at least partially sterilised. The destruction of bacteria by the current is readily shown with the new electro-microscopic slide of Dr. R. L. Watkins, New York.

PROFESSOR CORFIELD presided over a meeting of the General Committee of the Sunday Society in Prince's Hall, at which arrangements were made for opening to members the Exhibition of the Institute of Painters in Oil Colours on three Sundays before Christmas. The Committee also resolved to give concerts on these occasions, the first being on Sunday, December 6th, when the artistes will be Mdlle. Marie de Lido, Madame Amy Sandon, and Mr. Hirwen Jones, vocalists; Miss Marianne Eissler, violin; Miss Clara Eissler, harp; and Herr Carl Weber, solo pianist. Dr. Gritton, of the Lord's Day Observance Society, writes that "friendly notice" has been sent to persons who ventured to indulge is recreation on Sundays warning them of the risks they run, but he says that no communication of any kind had been made to the National Sunday League.

ALL hospital secretaries, and especially the secretaries of the Metropolitan hospitals, have an object lesson at the present time which they will, no doubt, take to heart. Unfortunately, journalism has always been hampered by prints which try to live by levying black-mail on the unwary and timid. The one idea of the conductors seems to be to impress upon the secretaries that these prints exist for the assistance of the charities on which they live. In practice, however, their columns prove that the one object of their existence is "to help the institutions (?)" by compelling the secretaries to advertise in their paper. The editors continuously preach, "If you do not advertise with us then you are a fool. If you do—no matter what else you may be—in our columns you will find yourselves depicted as angels of light and leading." Is it not about time that the secretaries put a stop to the nuisance by refusing to advertise at all in prints of the description referred to?

SURGEON-GENERAL SIR JOSEPH FAYRER, speaking at a meeting of the Funeral Reform Association in the Church House, Westminster, said that they had to deal with a most important subject, namely, the proper disposal of the dead. As the large centres of population became more condensed the question would assume much more importance, because of the danger to the public health arising from the improper disposal of the dead, whether by polluting the atmosphere and water, or by, if not engendering any special form of disease, at all events, rendering persons liable to disease. There was much to be said on the side of sentiment, which he would not touch on. The body was to be resolved into its original elments, and no matter how they resolved it, they obtained the same result. He agreed with Dr. Parkes, who stated: "In reality neither affection nor religion can be outraged by any manner of disposal of the dead, which is done with proper solemnity and respect to the earthly dwelling-place of our friends. The question should be placed entirely on sanitary grounds; and we then should judge it rightly." Sir Joseph said he was entirely in sympathy with the plans advocated by the society with regard to perishable coffins and a dry, porous earth. In no case should oaken coffins be used, and burial should be expedited as much as possible. There were certain cases in which cremation was necessary, namely, after a battle or during a pestilence, but as long as earth of the right sort was available, and the body enclosed in a perishable coffin, the absolute necessity was not apparent. A very great improvement had taken place in the way in which funerals were conducted, and he hoped that in the course of time the association would be successful in bringing about a further improvement in the burial of the dead.

